# Syntheses of Benzo[*d*]Thiazol-2(3*H*)-One Derivatives and Their Antidepressant and Anticonvulsant Effects

**DOI:** 10.3390/md17070430

**Published:** 2019-07-23

**Authors:** Qinghao Jin, Zhiyang Fu, Liping Guan, Haiying Jiang

**Affiliations:** 1 Donghai Science and Technology College, Zhejiang Ocean University, Zhoushan 316000, China; 2 Food and Pharmacy College, Zhejiang Ocean University, Zhoushan 316022, China; 3 College of Medicine, Jiaxing University, Jiaxing 314001, China

**Keywords:** benzo[*d*]thiazol, synthesis, antarctic-derived fungus, antidepressant, anticonvulsant

## Abstract

Thirty-four new benzo[*d*]thiazol derivatives **2a**–**2i**, **3a**–**3r**, and **4a**–**4g** were synthesized and investigated for their potential antidepressant and anticonvulsant effects. In a forced swimming test, **2c** and **2d** showed the highest antidepressant and anticonvulsant effects. **2c** and **2d** displayed a higher percentage decrease in immobility duration (89.96% and 89.62%, respectively) than that of fluoxetine (83.62%). In the maximal electroshock seizure test, **3n** and **3q** showed the highest anticonvulsant effect, with ED_50_ values of 46.1 and 64.3 mg kg^−^^1^, and protective indices of 6.34 and 4.11, respectively, which were similar to those of phenobarbital or valproate. We also found that the mechanism for the antidepressant activity of **2c** and **2d** may be via increasing the concentrations of serotonin and norepinephrine.

## 1. Introduction

During selective evolution, microorganisms in the polar region are distinct from other microorganisms with respect to genomic composition and have unique molecular–biological mechanisms, including metabolic regulation. Tan et al. reported very rich microbial resources in the Arctic and Antarctic [1]. In addition to extremely high species diversity [2], the structures of secondary metabolites of polar microbes also show diversity. Therefore, the Arctic and Antarctic regions are considered to be valuable natural products pools.

**Figure 1:**
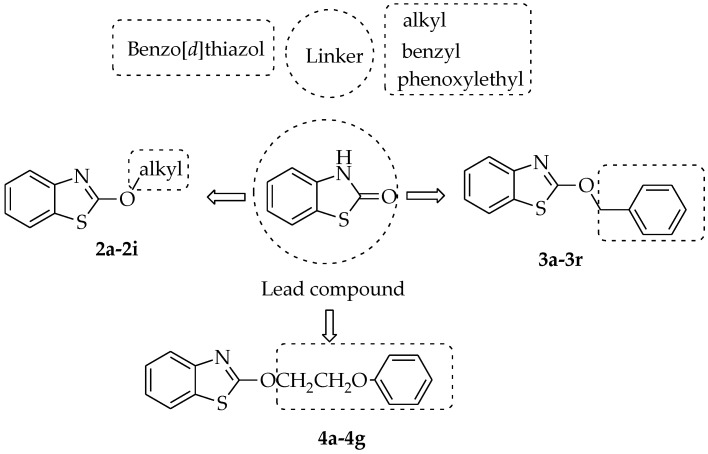
Structures of the lead compounds and derivatives **2a**–**2i**, **3a**–**3r**, and **4a**–**4g**.

In an investigation of the secondary metabolites of the Antarctic-derived fungus *Penicillium sp*. 44.42 °W, 60.54 °S, 239 m underwater, water temperature of −1.16 °C), Jiao et al. reported the isolation of nine compounds from the fermentation broth of *Penicillium sp*. [3], including benzo[*d*]thiazol-2(3*H*)-one (Figure 1). Benzothiazoles are heterocyclic aromatic hydrocarbons containing phenyl and thiazole rings, as well as sulfur and nitrogen atoms in their structures. Benzothiazole derivatives display a wide spectrum of pharmacologic effects, including anti-inflammatory [4], antibacterial [5], antiviral [6], antioxidant [7], and immunomodulatory properties [8]. In addition to the central nervous system (CNS)-related pharmacologic effects, benzothiazole compounds have been reported to display selective inhibitory effects against monoamine oxidase [9,10,11,12], as well as anti-Alzheimer’s disease [13] and convulsions [14]. The antidepressant effect of a series of benzothiazole derivatives has been demonstrated through animal models such as the tail suspension test (TST) and forced swimming test (FST) [15,16].

**Scheme 1:**
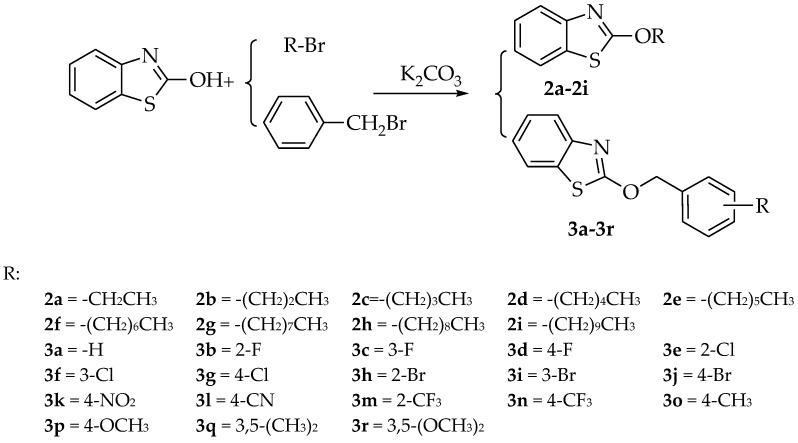
Synthesis of **2a**–**2i** and **3a**–**3r**.

**Scheme 2:**
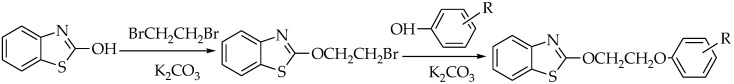

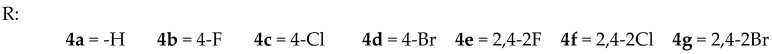
The synthetic route of derivatives **4a**–**4g**.

Several reports have described the antidepressant and anticonvulsant activities of benzothiazole derivatives [15,16]. For this reason, we synthesized thirty-four new benzo[*d*]thiazole derivatives **2a**–**2i**, **3a**–**3r** and **4a**–**4g** (Scheme 1 and Scheme 2) and investigated their potential antidepressant activity using the FST and their potential anticonvulsant effect using the maximal electroshock seizure (MES) test as well as their toxicity.

## 2. Results

### 2.1. Synthesis

Target compounds **2a**–**2i**, **3a**–**3r**, and **4a**–**4g** were prepared as shown in Scheme 1 and Scheme 2. Commercially available benzo[*d*]thiazol-2-ol was the starting material; the derivatives **2a**–**2i** and **3a**–**3r** were obtained through the introduction of an alkyl group or benzyl group by a one-step nucleophilic substitution reaction. Compounds **4a**–**4g** were obtained through a two-step reaction. The intermediate 2-(2-bromoethoxy)benzo[*d*]thiazole was synthesized and underwent a nucleophilic substitution reaction with 1,2-dibromoethane, then the 2-(2-bromoethoxy) benzo[*d*] thiazole formed was reacted with substituted phenols. The structures of **2a**–**2i**, **3a**–**3r**, and **4a**–**4g** were determined by infrared spectrophotometry, ^1^H and ^13^C NMR spectroscopy, as well as mass spectrometry.

### 2.2. Antidepressant Activity of ***2a***–***2i***, ***3a***–***3r***, and ***4a***–***4g*** in the FST

The antidepressant activity of fluoxetine, **2a**–**2i**, **3a**–**3r**, and **4a**–**4g**, as indicated by the immobility time in the FST, is displayed in Table 1. Most of the compounds, except for **3h**, **3k**, **3l**, **3r**, **4c**, and **4e**, induced a significant decrease in the immobility time at 30 mg kg^−1^ and showed a marked antidepressant effect (Table 1). In particular, **2b**–**2d**, **2f**, and **3q** possessed the highest antidepressant effect and induced a significant decrease in the immobility time compared with that of the control group (*p* < 0.001).

To better understand the antidepressant effect of **2a**–**2i**, **3a**–**3r**, and **4a**–**4g**, the percentage reduction in the time of immobility (% TID) was calculated using the formula

% TID = [(X − Y)/X] × 100

where X is the immobility time (s) for the control group and Y is the immobility time (s) for the test group. **2c**, **2d**, **2f**, and **3q** reduced the immobility time and showed higher TID values than the other tested compounds (Table 1). The % TID values for **2f** (85.10%) and **3q** (85.32%) were similar to that of fluoxetine at a concentration of 30 mg kg^−1^ in the FST. However, **2c** and **2d** showed higher % TID values (89.96% and 89.62%, respectively) than that of fluoxetine (83.62%), suggesting that these compounds may have superior antidepressant effects compared with that of fluoxetine (duration of immobility (s): **2c** = 18.0 ± 2.4; **2d** = 18.6 ± 6.8; fluoxetine = 31.8 ± 7.7).

### 2.3. Anticonvulsant Activity of ***2a***–***2i***, ***3a***–***3r***, and ***4a***–***4g*** in the MSE Test

The phase-I test study comprised two parts: MES and toxicity. The toxicity was measured by the rotorod toxicity experiment. Compounds **2a**–**2i**, **3a**–**3r**, and **4a**–**4g** were assessed for their anticonvulsant activity. The phase-I test was a qualitative analysis, with three doses of the test compounds administered (30, 100, and 300 mg kg^−1^). A protective effect was observed in mice through intraperitoneal administration of **2a**–**2i**, **3a**–**3r**, and **4a**–**4g** in the MES test (Table 2). Except for **2f**–**2i**, **3h**, **3j**, **3k, 4a** and **4f**, other derivatives displayed an anticonvulsant effect. Compounds **2a**, **3a**, **3l**, **3n**–**3q**, and **4b** displayed the highest anticonvulsant effect at 30 mg kg^−1^ in an MES test. Compounds **2c**–**2e**, **3b**, **3e**–**3g**, **3i**, **3m**, **3r**, **4d**, **4e** and **4g** were active at 100 mg kg^−1^. While **2b**, **3c**, **3d**, and **4c** showed the anticonvulsant activity at 300 mg kg^−1^. The rotorod toxicity experiment indicated that **2a**–**2i**, **3a**–**3r**, and **4a**–**4g** did not display toxicity at the test doses. In addition, all compounds were excreted or metabolized in ~4 h.

Next, the effect of **2a**, **3a**, **3b**, **3e**, **3l**, **3n**–**3q**, and **4b** were evaluated quantitatively for their anticonvulsant activity median effective dose (ED_50_) and neurotoxicity median toxicity dose (TD_50_) (Table 3) in a phase-II experiment. **3n** and **3q** showed the greatest effect, with ED_50_ values of 46.1 and 64.3 mg kg^−1^, and protective index (PI) of 6.34 and 4.11, respectively, which were higher than those of phenobarbital and valproate.

### 2.4. Effects of ***2c*** and ***2d*** on Monoamine Levels

Monoamine concentrations in the mouse brain are shown in Table 4. The concentration of **2c**, **2d,** and fluoxetine was 30 mg kg^−^^1^. The neurotransmitter concentration was calculated as ng g per brain region wet weight. **2c** and **2d** did not alter the dopamine concentration, but increased the concentrations of serotonin and norepinephrine in mouse brain significantly in the FST, and these effects were similar to those of the positive control fluoxetine.

## 3. Discussion

The FST is a model of depression. It mimics the condition of hopelessness and has a good predictive validity in mice. In this model, mice are limited in movement and cannot abscond, which results in motionlessness [18]. The immobility displayed in this model has been assumed to correspond to a behavioral response to hopelessness which, in turn, might correspond to a depressive disorder in humans [19].

Nine benzo[*d*]thiazole derivatives (**2a**–**2i**) containing an alkyl group with 2–10 carbons displayed antidepressant activity. Among them, **2c** (*n*-butyl group) and **2d** (*n*-pentyl group) exhibited the highest antidepressant activity. Although alkyl groups are not a functional group, they can play an important role in the binding interactions of a drug with its target. Alkyl chains are hydrophobic and can interact with the hydrophobic region of a receptor through Van der Waals interaction in the binding site. Varying the size of the group allows exploration of the hydrophobic region [20].

Among 18 benzyloxybenzo[*d*]thiazole derivatives **3a**–**3r**, most of the compounds, except for **3h**, **3k**, **3l** and **3r**, induced a significant decrease in the immobility time at 30 mg kg^−1^ and showed marked antidepressant effect. Interestingly, **3q**, which has two methyl substituents on the phenyl ring, displayed the highest antidepressant effect. 

However, the reduction conditions needed would be quite harsh and might not be feasible without causing drug degradation [20]. The position of halogen atoms affects antidepressant activity on the phenyl ring. 

Comparing the F-substituted compounds **3b**, **3c** and **3d** at different positions on the phenyl ring, the sequence of effect was 3-F > 4-F > 2-F, and the sequence of effect for Cl-substituted compounds **3e**, **3f**, and **3g** was 3-Cl > 4-Cl > 2-Cl. The sequence of effect for different Br-substituted compounds **3h**, **3i**, and **3j** was 3-Br > 4-Br > 2-Br. In addition, among the compounds with electron-withdrawing groups (i.e., **3k**, **3l**, **3m,** and **3n),** only **3m** and **3n** (with a –CF_3_ group) exhibited the antidepressant activities. For compounds with electron-donating groups, **3o–3r**, the sequence of activity was 3,5-(CH_3_)_2_ > 4-OCH_3_ > 4-CH_3_ > 3,5-(OCH_3_)_2_. Of seven phenoxylethoxylbenzo[*d*]thiazole compounds **4a**–**4g**, except for **4c** and **4e**, the remaining five compounds **4a**, **4b**, **4d**, **4f**, and **4g** induced a significant decrease in the immobility time at 30 mg kg^−^^1^ and exhibited antidepressant effects. Among them, **4a** displayed the highest antidepressant activity.

Nervous stress can cause impressionable individuals to develop epilepsy, and depressive illness is a general comorbidity related to epilepsy [21]. Nevertheless, understanding the heterogeneity of depression and epilepsy is difficult [22] Antiepileptic drugs might ameliorate the symptoms of depression, as indicated in clinical studies. Curing depression will have positive effects on epilepsy and quality of life. 

The anticonvulsant effects of **2a**–**2i**, **3a**–**3r**, and **4a**–**4g** were evaluated using the MES test. The most efficacious compounds, **3n** and **3q**, exhibited ED_50_ values of 46.1 and 64.3 mg kg^−1^ and had PI values of 6.34 and 4.11, respectively, which were greater than those of phenobarbital or valproate. Therefore, **3n** and **3q** might be useful candidates as antidepressant drugs for curing depression in patients with epilepsy.

A disruption in the release of neurotransmitters in the CNS, such as serotonin, norepinephrine, and dopamine, has been proposed to be a characteristic of depression. The metabolic imbalance of monoamine transmitters is considered to be a fundamental neurochemical feature in patients with depression. Hence, patients could be treated by increasing monoamine concentrations in the CNS [23]. We found that **2c** and **2d** increased concentrations of serotonin and norepinephrine markedly without altering dopamine concentrations in mouse brains, in a similar manner to that seen with the positive control fluoxetine in the FST. A cure for patients with major depression is deemed to include an increase in levels of serotonin or norepinephrine [24,25]. Thus, the antidepressant activities of **2c** and **2d** could be reflected by measuring levels of serotonin and norepinephrine in the CNS.

## 4. Method and Material

### 4.1. Reagents and Instruments

Positive drug: fluoxetine (purity > 99%) was purchased from Sigma. Melting points were measured by the melting point apparatus (WRS-1B, Shanghai, China). Infrared spectra (IR in KBr) were recorded using FT-IR1730 (Bruker, Switzerland). ^1^H and ^13^C NMR spectra were recorded on an AV-300 (Bruker, Switzerland), and the chemical shift values are in ppm relative to the TMS or solvent peaks. Mass spectra were recorded on MALDI-TOF/TOF mass spectrometer (Bruker Daltonik, Bremen, Germany). Main reagents were purchased from Aldrich Chemical Corporation (Shanghai, China). 

### 4.2. Synthesis of Benzo[d]thiazol and Benzyloxybenzo[d]thiazole Derivatives **2a**–**2i**, **3a**–**3r**

A solution of benzo[*d*]thiazol-2-ol (3.0 mmol), anhydrous K_2_CO_3_ (3.0 mmol) and 5 mL DMF was stirred in a round-bottomed flask for 1 h at 60 °C, then, 1.2 mmol of alkyl bromide or substituted brominated benzyl compound was added slowly to the reaction solution. The reaction solution was refluxed for 5 h, the reaction was monitored by TLC. DMF was evaporated under reduced pressure, the residue was washed with water, filtered, dried and the crude product was crystallized from MeOH. The yield, melting point, and spectral data of each compound are given below.

### 4.3. Synthesis of Ethoxylbenzo[d]thiazole Derivatives **4a**–**4g**

A mixture of benzo[*d*]thiazol-2-ol (3.0 mmol, 0.5 g), 1,2-dibromoethane (3.0 mmol, 0.6 g) and anhydrous K_2_CO_3_ (3.0 mmol, 0.4 g) was refluxed in DMF for 1 h, after the completion of the reaction (as monitored by TLC), DMF was evaporated and the precipitated product was washed with deionized water, dried. Then, 2-(2-bromoethoxy)benzo[*d*]thiazole (3.0 mmol, 0.8 g), 10 mL of a mixture of NaOH and substituted phenol was refluxed in EtOH for 2–5 h. After the completion of the reaction (as monitored by TLC), the solution was filtered and washed with 10% HCl and water. The crude product was recrystallized from MeOH. The melting points, yields, and spectral data of **4a**–**4g** are given below.

### 4.4. Experimental Animal and Compounds Treatment

Male ICR mice (20 ± 2 g) were purchased from the laboratory of animal study of Zhejiang Academy of medical sciences. Before the experiment started, mice were tamed for 1 week. During and before the test, mice were kept at 23 ± 2 °C for 12 h, at day and night circle, and tap water and standard food granules were provided. The procedures were adopted according to the National Institute of Health Guide for the Care and Use of Laboratory Animals and approved by the Ethics Committee of our Institution in this study. All the test compounds were dissolved in PEG-400 (polyethylene glycol-400). Other drugs were dissolved in 0.9% NaCl (isotonic saline solution). Fluoxetine, phenobarbital, and valproate were used as positive controls and the vehicle as the negative control. All the test compounds and other drugs were administered intraperitoneally for 30 min in the FST, the volume of the drug solution and vehicle was 0.1 mL/20 g of mice.

### 4.5. In the FST

Male ICR mice were randomized into groups. On the day of the experiment, mice were placed one at a time into a Perspex barrel (elevation 20 cm, 10 cm diameter) including 10 cm water about 22 °C. Mice were arranged into different groups (*n* = 8). Next, a mouse was placed independently into the perspex barrel and kept in the water for six minutes. After two minutes of fierce struggle, the mice were immobile. The duration of immobility was recorded during the last four min of the six min test. The immobility course was treated as the time that the mice floating on the water without struggle and maintained only the movements necessary to provide their head above the water [26,27].

### 4.6. In the MES Experiment

Convulsions were initiated in mice with a 60 Hz alternating current for 50 mA. The electric current was implemented via corneal electrodes for 0.3 s. Protection against the spread of the maximal electroshock seizure-induced seizures was defined as the abolition of the hind leg and tonic maximal extension component of the seizure. At 30 min after the administration of the compounds, the activity was evaluated in the maximal electroshock seizure test [28].

### 4.7. Experiment of Neurotoxicity 

The neurotoxicity experiment of the compounds and drugs was evaluated through the rotorod experiment in mice. The mice were trained to stay on an accelerating rotorod of diameter 3.2 cm that rotated at 10 rpm. Trained animals were given an intraperitoneal injection of the test compounds. Neurotoxicity was indicated by the inability of the animal to maintain equilibrium on the rod for at least 1 min in each of the trials. The MES and rotorod tests were carried out according to the standard procedure described in the Antiepileptic Drug Development Program (ADD) of the National Institutes of Health (USA) [29].

### 4.8. HPLC conditions and Sample Preparation

The dosage of 30 mg kg^−^^1^ of **2b**, **2c** and fluoxetine was used for testing the action on MOA neurochemical levels in rat brain. Mice were randomly divided into five groups (*n* = 10). **2b**, **2c** and fluoxetine, normal vehicle, stress vehicle oral gavage once a day for seven days. After the end of the test, the mouse was immediately sacrificed by cervical dislocation, then the brain tissue was immediately removed, and quickly frozen and at −80 °C until used for neurochemical analysis. The brain tissues were sonicated in 0.1 M NaH_2_PO_4_ aqueous solution including 0.85 mM OSA, 0.5 mM Na_2_∙EDTA (ethylenediamine tetraacetic acid disodium), centrifuged at 13,000× *g* at 4 °C for 15 minutes. Then serotonin, norepinephrine and dopamine were analyzed by High-Performance Liquid chromatography coupled with an electron capture detector. The mobile phase was made up of 0.1 mol L^-1^ anhydrous sodium dihydrogen phosphate containing 0.5 mM EDTA and 0.85 mM osanetant (OSA) and 11% MeOH and regulated to pH 3.4 using phosphate acid buffer solution and filtered by the pore size ultrafiltration membrane of 0.45 μM. The external standard curves were used to quantify the amounts of serotonin, noradrenaline, and dopamine in each sample calculated by area under the curve. The injection volume dose was 20 μL. The detection limit of the analysis was 20 pg∙g^−^^1^ sample. 

### 4.9. Statistic Analysis

All analyses were performed using the GraphPad Prism program (GraphPad software, Inc., San Diego, CA, USA). The statistical analysis of the behavioral tests was performed by analysis of variance (ANOVA), which was followed by Tukey’s post hoc comparison test. All experimental results are presented as mean (s) ± standard error of the mean (SEM), with a *p*-value smaller than 0.05 considered statistically significant.

## 5. Conclusions

Thirty-four previously unreported benzo[*d*]thiazol derivatives **2a**–**2i**, **3a**–**3r**, and **4a**–**4g** were prepared and assessed for their potential antidepressant and anticonvulsant effects. **2c** and **2d** decreased the immobility time markedly and displayed the highest antidepressant effects in the FST, and also showed anticonvulsant activity. **2c** and **2d** did not change the dopamine concentration but increased the concentrations of serotonin and norepinephrine significantly in the mouse brain in the FST, similar to that observed with the positive control fluoxetine. These results suggest that **2c** and **2d** may be potential leads for the development of therapeutic agents for the treatment of depression and epilepsy.

## Figures and Tables

**Table 1:** Antidepressant effect of **2a**–**2i**, **3a**–**3r**, and **4a**–**4g**.

Antidepressant Effect		
Compounds	Duration of Immobility (s)	TID ^a^ (%)
**2a**	122.5 ± 9.2 *	31.64
**2b**	59.3 ± 8.8 ***	66.91
**2c**	18.0 ± 2.4 ***	89.96
**2d**	18.6 ± 6.8 ***	89.62
**2e**	104.2 ± 9.7 *	46.32
**2f**	26.7 ± 7.4 ***	85.10
**2g**	67.7 ± 10.7 **	62.22
**2h**	76.0 ± 13.2 **	57.59
**2i**	93.0 ± 9.0 **	48.10
**3a**	81.2 ± 9.4 **	54.69
**3b**	84.8 ± 5.6 **	52.68
**3c**	61.5 ± 3.3 **	65.68
**3d**	75.2 ± 8.2 **	58.04
**3e**	132.2 ± 8.5 *	26.23
**3f**	68.2 ± 11.5 **	61.94
**3g**	80.3 ± 11.9 **	55.19
**3h**	141.3 ± 8.5	21.15
**3i**	66.0 ± 8.1 **	63.17
**3j**	99.3 ± 8.7 **	44.59
**3k**	153.8 ± 11.0	14.17
**3l**	172.5 ± 5.4	3.74
**3m**	125.8 ± 13.6 *	29.80
**3n**	124.5 ± 14.3 *	30.52
**3o**	130.6 ± 5.4 *	27.12
**3p**	71.2 ± 7.0 **	60.27
**3q**	26.3 ± 10.3 ***	85.32
**3r**	143.2 ± 11.4	20.09
**4a**	67.5 ± 7.3 **	62.33
**4b**	130.5 ± 7.9 *	37.56
**4c**	141.7 ± 9.1	32.20
**4d**	116.2 ± 7.0 *	44.40
**4e**	151.7 ± 14.1	27.42
**4f**	129.5 ± 5.4 *	38.04
**4g**	124.2 ± 8.1 *	40.57
control	194.1 ± 11.1	-
fluoxetine	31.8 ± 7.7 ***	83.62

^a^ TID: percentage decrease in immobility duration. Significant differences versus control values. * *p* < 0.05, ** *p* < 0.01, *** *p* < 0.001.

**Table 2:** Anticonvulsant activity of **2a**–**2i**, **3a**–**3r**, and **4a**–**4g** in a phase-I study.

Compounds	Dosage (mg∙kg^−^^1^)	MES ^a^		Rotorod ^b^	
		0.5 h	4 h	0.5 h	4 h
**2a**	30	1/3	0/3	0/3	0/3
**2b**	300	1/3	0/3	0/3	0/3
**2c**	100	1/3	0/3	0/3	0/3
**2d**	100	1/3	0/3	0/3	0/3
**2e**	100	1/3	0/3	0/3	0/3
**2f**	300	0/3	0/3	0/3	0/3
**2g**	300	0/3	0/3	0/3	0/3
**2h**	300	0/3	0/3	0/3	0/3
**2i**	300	0/3	0/3	0/3	0/3
**3a**	30	1/3	0/3	0/3	0/3
**3b**	100	3/3	0/3	0/3	0/3
**3c**	300	1/3	0/3	0/3	0/3
**3d**	300	1/3	0/3	0/3	0/3
**3e**	100	3/3	0/3	0/3	0/3
**3f**	100	1/3	0/3	0/3	0/3
**3g**	100	1/3	0/3	0/3	0/3
**3h**	300	0/3	0/3	0/3	0/3
**3i**	100	1/3	0/3	0/3	0/3
**3j**	300	0/3	0/3	0/3	0/3
**3k**	300	0/3	0/3	0/3	0/3
**3l**	30	2/3	0/3	0/3	0/3
**3m**	100	2/3	0/3	0/3	0/3
**3n**	30	3/3	0/3	0/3	0/3
**3o**	30	1/3	0/3	0/3	0/3
**3p**	30	2/3	0/3	0/3	0/3
**3q**	30	3/3	0/3	0/3	0/3
**3r**	100	2/3	0/3	0/3	0/3
**4a**	300	0/3	0/3	0/3	0/3
**4b**	30	1/3	0/3	0/3	0/3
**4c**	300	1/3	0/3	0/3	0/3
**4d**	100	1/3	0/3	0/3	0/3
**4e**	100	1/3	0/3	0/3	0/3
**4f**	300	0/3	0/3	0/3	0/3
**4g**	100	1/3	0/3	0/3	0/3
**Valproate**	100	3/3	0/3	0/3	0/3

^a^ MES test (numbers of mice defended/numbers of mice detected); ^b^ Toxicity: rotorod test (numbers of mice showing toxicity/numbers of mice detected).

**Table 3:** Anticonvulsant activity of **2a**, **3a**, **3b**, **3e**, **3l**, **3n–3q** and **4b** in a phase-II study.

Compounds	ED_50_^a^ (mg/kg)	TD_50_^b^ (mg/kg)	PI (TD_50_/ED_50_)
**2a**	90.8	>200	3.20
**3a**	105.9	>200	2.89
**3b**	>300	>200	1.89
**3e**	147.2	>200	2.36
**3l**	>300	>200	2.04
**3n**	46.1	>200	6.34
**3o**	84.3	>200	3.37
**3p**	>300	>200	1.13
**3q**	64.3	>200	4.11
**4b**	74.5	>200	3.69
Phenobarbital ^c^	21.8	69.0	3.2
Valproate	247	>200	1.6

^a^ ED_50_: Median effective dose affording anticonvulsant defense for 50% animals; ^b^ TD_50_: Median toxic dose eliciting minimal neurological toxicity for 50% animals; ^c^ data from Krall et al. 1978 [17].

**Table 4:** Effect of **2c** and **2d** on monoamine concentrations in mouse brain.

Groups	Serotonin	Norepinephrine	Dopamine
Normal Vehicle	325.1 ± 28.3	298.4 ± 22.4	357.4 ± 29.8
Stress Vehicle	202.4 ± 38.4 ^c^	207.3 ± 25.7 ^c^	218.7 ± 20.0
**2c**	334.5 ± 31.9 ^b^^,^^c^	309.5 ± 20.6 ^a^^,^^c^	201.5 ± 19.2
**2d**	329.0 ± 27.8 ^b^^,^^c^	310.7 ± 24.9 ^a^^,^^c^	206.0 ± 18.7
Fluoxetine	340.3 ± 32.5 ^b^^,^^c^	321.8 ± 29.1 ^a^^,^^c^	202.6 ± 17.8

Values are given as mean ± SEM (*n* = 8). ^a^ *P* < 0.05, ^b^ *P* < 0.01 vs. stress vehicle; ^c^ *P* < 0.05 vs. normal vehicle.
